# *Toxoplasma* sortilin interacts with secretory proteins and it is critical for parasite proliferation

**DOI:** 10.1186/s13071-024-06207-7

**Published:** 2024-03-04

**Authors:** Chenghuan Li, Ning Jiang, Yize Liu, Yiwei Zhang, Ran Chen, Ying Feng, Xiaoyu Sang, Qijun Chen

**Affiliations:** 1 Key Laboratory of Livestock Infectious Diseases, Ministry of Education, and Key Laboratory of Ruminant Infectious Disease Prevention and Control (East), Ministry of Agriculture and Rural Affairs, College of Animal Science and Veterinary Medicine, Shenyang Agricultural University, 120 Dongling Road, Shenyang, 110866 China; 2 Research Unit for Pathogenic Mechanisms of Zoonotic Parasites, Chinese Academy of Medical Sciences, 120 Dongling Road, Shenyang, 110866 China

**Keywords:** *Toxoplasma gondii*, Sortilin, Vps10, Bio-layer interferometry, AF38469, Drug target

## Abstract

**Background:**

The human sortilin protein is an important drug target and detection marker for cancer research. The sortilin from *Toxoplasma gondii* transports proteins associated with the apical organelles of the parasite. In this study, we aimed to determine the intracellular localization and structural domains of *T. gondii* sortilin, which may mediate protein transportation. Approaches to the functional inhibition of sortilin to establish novel treatments for *T. gondii* infections were explored.

**Methods:**

A gene encoding the sortilin protein was identified in the *T. gondii* genome. Immunoprecipitation and mass spectrometry were performed to identify the protein species transported by *T. gondii* sortilin. The interaction of each structural domain of sortilin with the transported proteins was investigated using bio-layer interferometry. The binding regions of the transported proteins in sortilin were identified. The effect of the sortilin inhibitor AF38469 on the infectivity of *T. gondii* was investigated. The binding site of AF38469 on sortilin was determined.

**Results:**

The subdomains Vps10, sortilin-C, and sortilin-M of the sortilin were identified as the binding regions for intracellular transportation of the target proteins. The sortilin inhibitor AF38469 bound to the Vps10 structural domain of *T. gondii* sortilin, which inhibited parasite invasion, replication, and intracellular growth in vitro and was therapeutic in mice infected with *T. gondii*.

**Conclusion:**

The Vps10, sortilin-C, and sortilin-M subdomains of *T. gondii* sortilin were identified as functional regions for intracellular protein transport. The binding region for the sortilin inhibitor AF38469 was also identified as the Vps10 subdomain. This study establishes sortilin as a promising drug target against *T. gondii* and provides a valuable reference for the development of anti-*T. gondii* drug-target studies.

**Graphical Abstract:**

**Figure:**
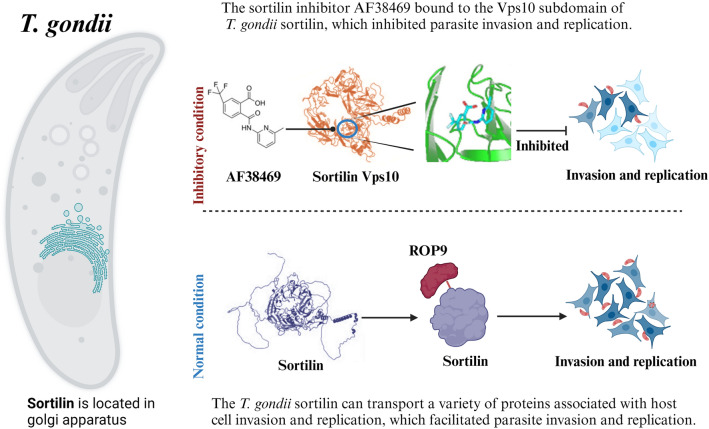

**Supplementary Information:**

The online version contains supplementary material available at 10.1186/s13071-024-06207-7.

## Background

*Toxoplasma gondii*, a specialized intracellular parasite of the Apicomplexa, can actively invade all nucleated cells of warm-blooded animals [1, 2]. Toxoplasmosis is an important infectious disease in immunocompromised patients, affecting approximately 30% of the global population [2]. Therefore, diseases caused by *T. gondii* pose serious threats to human health and global livestock production [3]. *Toxoplasma gondii* is a unicellular intracellular parasite with an endoplasmic reticulum (ER) and Golgi apparatus, as well as specialized secretory organelles, such as micronemes, rhoptries, and dense granules [3]. Upon invasion of host cells, these organelles secrete proteins with various functions to assist tachyzoites in entering host cells and forming a parasitophorous vacuole (PV) to replicate by binary division [2, 4]. During invasion, *T. gondii* first recognizes host cells through surface antigen proteins (SAGs) [5], which allow the apical secretory organelles of *T. gondii* to adhere to the host cell surface [6–8]. Subsequently, micronemes secrete microneme proteins (MICs), which can promote mutual recognition between *T. gondii* and the host surface-related receptors [9], identify suitable invasion sites to advance parasite invasion, and assist in the formation of the moving junction (MJ) structure [10–12]. Subsequently, the rhoptry organelles secrete rhoptry proteins (ROPs) and rhoptry neck proteins (RONs) [13]. RONs contribute to the formation of the MJ structure, which promotes *T. gondii* invasion of host cells. ROPs can manipulate host cell pathways, including those that control innate immunity and are involved in PV formation, thus contributing to invasion and proliferation [14]. After *T. gondii* enters host cells, dense granule proteins (GRAs) promote PV formation, which is critical for proliferation [15–17]. These proteins, which are virulence factors of the parasite, all require ER and Golgi trafficking to the secretory organelles [3, 18]. MIC, ROPs/RONs, and GRAs all depend on their N-terminal domain signal peptides to be transported from the ER-Golgi system or trans-Golgi network to the corresponding organelles in soluble or aggregated complexes [16, 19–23].

Sortilin (also known as SORT1) was first discovered in the human brain and is highly expressed in neurons; however, its expression in hepatocytes, adipocytes, and macrophages has also been identified [24]. As a type I membrane glycoprotein in the vacuolar protein sorting 10 (Vps10) domain receptor family, sortilin is also known as neurotensin receptor-3 (NTSR-3) [25]. Studies have shown that sortilin is a highly conserved membrane protein that acts as a receptor in extracellular and endocytic pathways to regulate protein trafficking [26, 27]. In the neurons of the human nervous system, sortilin was originally thought to be a special receptor, similar to sortilin in yeast cells [25]. Sortilin has been shown to play an important role in cell trafficking and signaling as a specific receptor for neurotrophic factors and neuropeptides and as a coreceptor for cytokine receptors, tyrosine kinase receptors, G protein-coupled receptors, and ion channels [25]. Furthermore, recent studies have identified sortilin as a potential cancer marker and an excellent drug target. In parasitic studies, *Plasmodium falciparum* sortilin has been found to transport relevant proteins from the cytoplasm to the corresponding apical secretory organelles. Sortilin knockdown reduces the growth and replication capacity of *P. falciparum* [28, 29]. In one study, *T. gondii* sortilin (TgSORT, 962 aa) was also shown to transport ROP and MIC families to secretory organelles. Knockdown of TgSORT significantly reduced parasite growth, invasion, and replication capacity, rendering gene-deleted parasites non-pathogenic to mice [30].

In this study, the sortilin protein of *T. gondii* (1033 aa, ~ 120 kDa) was found to be involved in the transport of several virulence factors of the parasite, such as SAGs and secreted proteins of apical organelles, including ROPs and MICs. Molecular interaction experiments using bio-layer interferometry (BLI) technology confirmed that the Vps10, sortilin-C, and sortilin-M subdomains of sortilin were the binding regions of the transported proteins. The sortilin inhibitor AF38469, originally found to reduce the invasiveness of pancreatic cancer cells and glioblastoma cells [31, 32], can also inhibit *T. gondii* cell invasion, replication in vitro, and infectivity in mice.

## Methods

### Cultivation of *T. gondii*

*Toxoplasma gondii* tachyzoites of both the RH and ME49 strains were cultured in DMEM/high-glucose medium (Servicebio, Wuhan, China) containing 10% fetal bovine serum (FBS) and incubated with Vero cells in an environment of 37 °C with 5% CO_2_.

### Bioinformatic analysis of sortilin

Sortilin sequences were obtained from the ToxoDB database (http://www.toxodb.org/toxo/). The structural domain of sortilin was predicted using the National Center for Biotechnology Information website (https://www.ncbi.nlm.nih.gov/). Signal peptides and transmembrane regions within protein sequences were predicted using SignalP (http://www.cbs.dtu.dk/services/SignalP/) and TMHMM (http://www.cbs.dtu.dk/services/TMHMM/) (Additional file 1: Fig. S1A). To investigate the protein function of sortilin, the machine learning method AlphaFold2 (https://github.com/deepmind/alphafold) was used to predict its 3D structure and compare it with that of murine and human sortilins in the database (Additional file 1: Fig. S1B–D).

### Generation of recombinant sortilin and subdomains

The protein-coding region of the sortilin gene (gene number: TGGT1_290160) was obtained from DNA isolated from *T. gondii* RH tachyzoites by PCR using gene-specific primers (Additional file 1: Table. S1). The amplicon was cloned into pET-28a and pGEX-4T-1 vectors (Invitrogen, Carlsbad, CA, USA). The two recombinant plasmids were expressed in *Escherichia coli* BL21 (DE3) cells (TransGen Biotech, Beijing, China). His- and GST-tagged recombinant proteins were obtained by affinity purification, as described previously [33]. Purified recombinant proteins were detected by Western blotting using specific antibodies against HIS and GST tags (Additional file 1: Fig. S2).

Four subdomain coding regions, named sortilin-N (1–166 aa), Vps10 (160–667 aa), sortilin-C (665–848 aa), and sortilin-M (880–1033 aa), were obtained using specific primers (Additional file 1: Table. S1) as described above. The four amplified gene fragments were cloned into the pGEX-4T-1 vector and expressed in *E. coli* BL21 cells (DE3). Finally, recombinant GST proteins were obtained by affinity purification using Glutathione Sepharose 4 B (Cytiva, Logan, UT, USA) (Additional file 1: Fig. S3A, B), as previously described [33].

### Generation of specific antibodies to *T. gondii* sortilin

*Toxoplasma gondii* sortilin-specific polyclonal antibodies were obtained by immunizing mice and rats with His-tagged sortilin recombinant proteins. Eight female BALB/c mice and three female Sprague-Dawley rats were subcutaneously immunized four times with Freund’s adjuvant. Each mouse was immunized subcutaneously with 50 μg of the HIS-tagged recombinant protein each time, and each rat was immunized subcutaneously with 100 μg of the recombinant protein each time. Blood was collected from the hearts of the rats and eyes of the mice. Blood samples were incubated at 37 °C for 1 h and then centrifuged at 3500 rpm for 10 min to separate the sera. All sera were inactivated at 56 °C for 30 min. Rat and mouse anti-sortilin IgGs were purified from serum using Protein G Sepharose 4 Fast Flow (GE Healthcare, Chicago, IL, USA), as described previously [33].

### Expression analysis of *T. gondii* sortilin

To investigate the expression of native *T. gondii* sortilin, 3 × 10^6^ purified tachyzoites from RH and ME49 strains were lysed. The lysates were dissolved in 5× SDS loading buffer (Beyotime, Shanghai, China), electrophoresed on a 10% SDS-PAGE gel, and transferred onto a PVDF membrane (Millipore, Boston, MA, USA). The membranes were blocked with 10 ml PBS containing 5% skim milk for 1 h at 37 °C and then incubated 13 h at 4 °C in PBST containing sortilin-specific rat IgG (1:800). After three washes with PBST, the membranes were incubated with alkaline phosphatase (AP)-conjugated goat anti-rat IgG (1:1000; EASYBIO, Beijing, China) at 37 °C for 1 h. The membranes were then washed three times with PBST and developed for 2 min using a BCIP/NBT AP chromogenic kit (Beyotime). Finally, the membranes were scanned using an imaging scanner.

### Intracellular localization of *T. gondii* sortilin with immunofluorescent assays

To localize *T. gondii* sortilin, 1 × 10^6^ purified tachyzoites were fixed in 4% paraformaldehyde (PFA; Beyotime) and permeabilized on slides using 0.25% Triton X-100 (Beyotime). The parasites were blocked with 1 ml PBS containing 5% skim milk for 30 min at 37 °C and incubated 13 h with rat anti-sortilin antibody at 4 °C (1:100). The slides were then washed four times with PBS and incubated with an Alexa Fluor 488-conjugated goat anti-rat secondary fluorescent antibody (1:1000; Invitrogen) for 1 h at 37 °C. After five washes with PBS, the slides were stained with DAPI (Invitrogen) for 10 min at 25 °C and washed five times with PBS. Parasites without permeabilization were examined in a similar manner. The samples were examined using a confocal laser scanning microscope (Leica SP8, Wetzlar, Germany).

In another experiment, rat anti-sortilin serum (1:100) and anti-GROASP antibody (1:200; EASYBIO) were co-incubated with 1 × 10^6^ parasites, and the fluorescence signals were recorded in the same way as described above. GROASP is a specific marker of the Golgi apparatus.

To further confirm the localization of sortilin in the Golgi apparatus of *T. gondii*, purified tachyzoites were treated with 5 μg/ml Brefeldin A (BFA; Beyotime), which specifically disrupts the Golgi apparatus, for 4 h in an incubator at 37 °C. The treated parasites (1 × 10^6^) were invaded into Vero cells in six-well plates for 4 h. The same number of parasites not treated with BFA was incubated with Vero cells in the same manner as the controls. Non-invasive *T. gondii* tachyzoites were washed away with PBS. The sortilin protein was examined with an immunofluorescence assay with protein-specific antibodies, as described above.

### Immunoprecipitation of proteins interacting with *T. gondii* sortilin

Purified tachyzoites (3 × 10^8^) of the *T. gondii* RH strain were lysed by sonication, and the total protein concentration was diluted to 10 μg/μl. Next, 200 μl of rat anti-sortilin-IgG (1 mg/ml), 500 μl of total parasite proteins, and 10 μl protease inhibitors (Beyotime) were thoroughly mixed and incubated at 4 °C for 13 h. Then, 100 μl of pre-washed protein G agarose beads (GE Healthcare) was added to the mixture and incubated at 4 °C with slow shaking for 4 h. The beads were washed five times in cold PBS, centrifuged at 4000 rpm for 10 min at 4 °C, resuspended with 80 μl PBS, mixed with 20 μl 5× SDS loading buffer, and boiled for 10 min. The samples were centrifuged at 4000 rpm for 10 min at 4 °C. The supernatants were then transferred to new tubes.

The samples were dissolved in 10% SDS-PAGE gels, and the protein bands were cut off for mass spectrometry analysis (Novogene Co. Ltd). Based on the results of mass spectrometry, sortilin-specific mouse IgG (1:800), TgROP9-specific mouse IgG (1:800), TgMIC3-specific mouse IgG (1:800), and TgSAG2-specific mouse IgG (1:800) were used as primary antibodies. Western blot analysis was performed as described above. *Toxoplasma gondii* total proteins were used as a positive control. Vero cell total proteins were used as a negative control. Finally, the membranes were imaged.

### Co-localization of sortilin with TgROP9, TgMIC3, and TgSAG2 in *T. gondii*

To co-localize TgROP9, TgMIC3, and TgSAG2 with TgSAG2, rat anti-sortilin antibody (1:100), mouse anti-TgROP9 antibody (1:100), mouse anti-TgMIC3 antibody (1:100), and mouse anti-TgSAG2 antibody (1:100) were used as primary antibodies. Alexa Fluor 488-conjugated goat anti-mouse antibody (1:1000) and Alexa Fluor 594-conjugated goat anti-rat antibody (1:1000) were used as secondary antibodies. Immunofluorescence analysis was performed as described above.

### Analysis of the molecular interaction of sortilin and subdomains with target proteins by BLI

The molecular interactions of sortilin with the target proteins were analyzed using the Ni–NTA sensor of a molecular interaction analyzer (Fortebio, Silicon Valley, CA, USA). The recombinant proteins His-TgROP9, His-TgMIC3, and His-TgSAG2 (1 μM) (Additional file 1: Fig. S3C) were used as binders and placed in 96-well plates. The concentrations of the recombinant GST-tagged sortilin protein were set to 100, 150, 200, 250, and 300 nM and placed in a 96-well plate, while the GST protein (1 μM) was used as a negative control. The reagents added to a 96-well plate from left to right were PBS, binders (His-tagged recombinant proteins), analytes/negative controls (GST-tagged recombinant proteins/GST protein), glycine, and nickel sulfate. Glycine and nickel sulfate were mainly used in the regeneration and neutralization cycles of the Ni–NTA sensor for ease of reuse. First, the Ni–NTA sensors were equilibrated in PBS for 60 s, and then recombinant His-TgROP9, His-TgMIC3, and His-TgSAG2 were added and incubated for 180 s. All sensors were washed in PBS for an additional 60 s. One group of sensors was incubated with different concentrations of GST-tagged recombinant proteins for 180 s, and another group was incubated with the GST control protein for 180 s. Finally, the sensors were placed in PBS to dissociate for 180 s, followed by three regeneration and neutralization cycles of 30 s each.

Following the protocol described above, the molecular interactions between sortilin subdomains and target proteins were further analyzed. His-tagged recombinant proteins were used as binders (1 μM). Sortilin subdomains, including GST-sortilin-N, GST-Vps10, GST-sortilin-C, and GST-sortilin-M recombinant proteins, were diluted to 200, 400, 600, 800, and 1000 nM. The GST protein (1 μM) was used as a negative control. The Ni–NTA sensors were equilibrated in PBS for 60 s and incubated with His-tagged recombinant proteins for 120 s. The sensors were then washed with PBS for 80 s. One group of sensors was incubated with different concentrations of GST-tagged recombinant proteins for 200 s, and another group was incubated with the GST control protein for 200 s. Finally, the sensors were placed in PBS to dissociate for 200 s, followed by three regeneration and neutralization cycles of 30 s each.

### Analysis of the effect of the sortilin inhibitor on *T. gondii* on host cell invasion and intracellular proliferation

AF38469, a sortilin inhibitor, was purchased from MedChem Express (Monmouth Junction, NJ, USA). Dimethyl sulfoxide (DMSO; Thermo Fisher Scientific, Waltham, MA, USA) was used to dissolve AF38469. We tested the effect of sortilin inhibitors on *T. gondii* invasion. Six concentration gradients of AF38469 (0, 75, 150, 300, 500, and 1000 nM) were added to 1 × 10^6^ purified parasites and incubated for 4 h at 37 °C, with the same concentration (v/v) of DMSO used as the no-treatment control. The invasion rates of cells treated with different concentrations of AF38469 were analyzed using a red-green assay [33]. AF38469-treated parasites (1 × 10^6^) were incubated in 12-well plates with Vero cells for 1 h. Parasites that did not invade the cells were washed away with PBS, and the cells were fixed with 4% PFA at 25 °C for 15 min, followed by three washes with PBS and blocking with 1 ml of 5% skim milk for 30 min. A rat anti-TgSAG2 antibody (1:100) was added to the cells and incubated for 1 h at 37 °C, followed by five washes with PBS. Then, 0.25% Triton X-100 was added to permeabilize the cells for 15 min at 25 °C, and the cells were washed three times with PBS. The cells were further incubated with mouse anti-TgROP9 antibody (1:100) for 1 h at 37 °C and washed five times with PBS. Alexa-594-conjugated goat anti-rat (1:1000; Invitrogen) and Alexa-488-conjugated goat anti-mouse (1:1000; Invitrogen). Secondary antibodies were added and incubated for 1 h. Finally, 10 fields were selected under a fluorescence microscope to observe differently colored parasites and counted [33, 34].

To test the effect of sortilin inhibition on the intracellular proliferation of *T. gondii*, 1 × 10^6^ AF38469-treated parasites was added to Vero cells in 12-well plates and incubated for 4 h. The non-invading parasites were washed away with PBS. After incubation for 24 h, the number of vacuoles containing 2, 4, 8, and 16 *T. gondii* tachyzoites was counted by fluorescence microscopy as described above, and 100 vacuoles were counted.

Caspimycin A23187 (Beyotime) increases intracellular Ca^2+^ levels and induces cell activation, differentiation, and proliferation. A23187 has often been used to induce *T. gondii* egress from host cells [35, 36]. In this study, we investigated the effects of A23187 on AF38469-treated *T. gondii*. AF38469-treated parasites (1 × 10^6^) were added to Vero cells in 12-well plates and incubated for 4 h. The non-invading parasites were washed with PBS. After 24 h of incubation, 1 ml of DMEM medium containing 5 μM A23187 was added to the plates for 20 min at 37 °C. The egress rate of *T. gondii* was calculated by observing 100 vacuoles using fluorescence microscopy, as described above.

The intracellular growth of *T. gondii* at different AF38469 concentrations was analyzed using a plaque assay. Vero cells were divided into six groups, each infected with 50 *T. gondii* tachyzoites, and incubated with the inhibitor AF38469 for 7 days at 37 °C. The culture medium containing AF38469 was changed daily. After 7 days, the plates were stained with 1 μM crystal violet (Beyotime) for 20 min. Images were captured to observe the intracellular growth of *T. gondii* at different concentrations of AF38469.

To determine the therapeutic effects of AF38469 on toxoplasmosis, 40 female BALB/c mice were divided into four groups. Each mouse was infected intraperitoneally with 100 *T. gondii* tachyzoites. The experimental group was orally administered 500 nM AF38469; PBS and DMSO were used as controls. The mice were observed for 15 days, and the survival rate of each group was calculated.

### Characterization of the binding site of the AF38469 inhibitor on *T. gondii* sortilin

The binding region of the AF38469 inhibitor to sortilin was identified using the BLI system. Sortilin has four structural domains: sortilin-N, Vps10, sortilin-C, and sortilin-M. The GST-tagged recombinant proteins were diluted to 1 μM and biotinylated with EZ-Link NHS-PEG12-Biotin (A35389, Thermo Fisher Scientific) for 45 min at 25 °C. The AF38469 inhibitor was diluted to 100, 200, 300, 400, or 500 nM in DMSO. The SSA sensors (Fortebio) were equilibrated in PBS for 60 s, loaded with recombinant sortilin-N, Vps10, sortilin-C, and sortilin-M for 120 s and washed with PBS for 90 s. The SSA sensors were then incubated with the AF38469 inhibitor at various concentrations for 120 s. Finally, the SSA sensors were dissociated in PBS for 300 s. DMSO was used as a negative control.

The machine learning method AlphaFold2 was used to predict the 3D simulated spatial structure of the sortilin protein and to produce simulated binding images of sortilin and AF38469. Specific binding sites of sortilin with AF38469 were also predicted.

### Statistical analysis

Analyses were performed using a molecular interaction analyzer (Fortebio). All data were analyzed using GraphPad Prism 5.0 (GraphPad Software, Inc., USA). The mean and standard deviation (SD) were determined using three biological replicates. Statistical thresholds of *P* < 0.05 and *P* < 0.01 were considered significant.

## Results

### The *T. gondii* sortilin was localized to the Golgi apparatus of tachyzoites

**Fig. 1:**
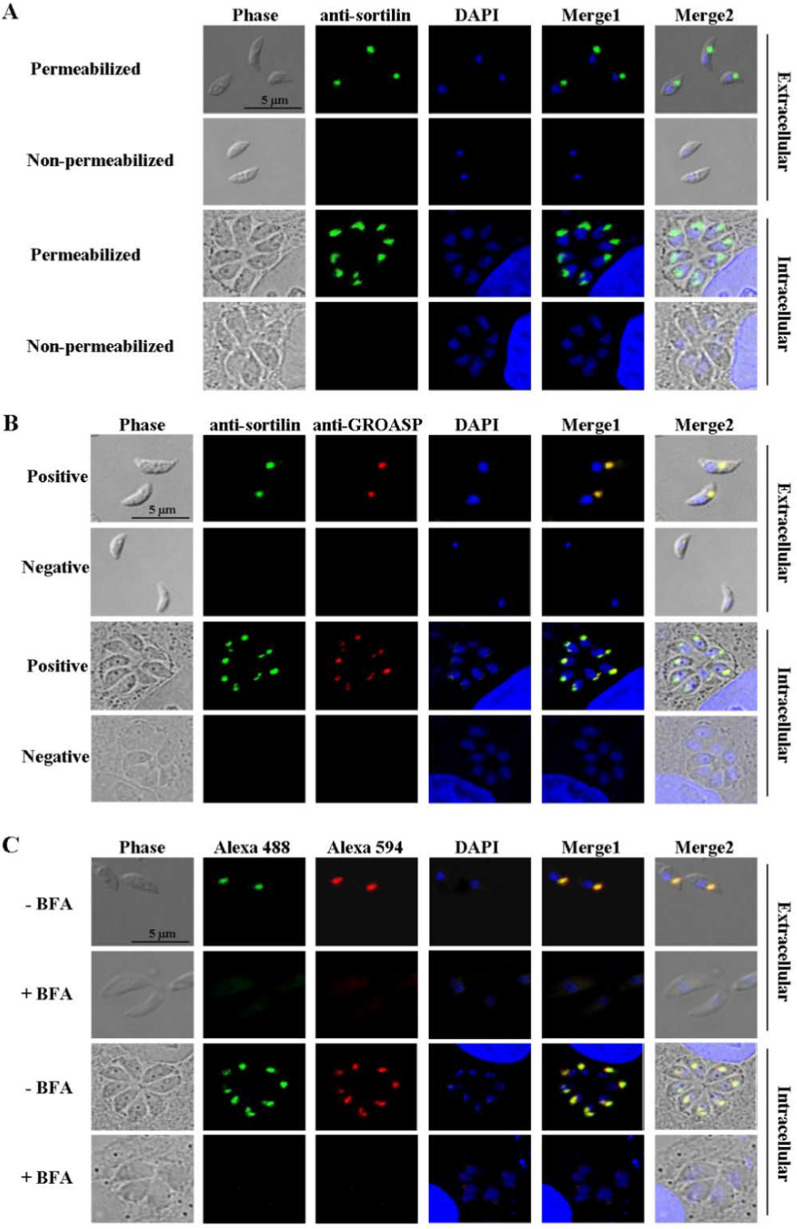
*Toxoplasma gondii* sortilin was localized in the Golgi apparatus. **A** After permeabilization with 0.25% Triton X-100, the sortilin fluorescence signal (green) was localized near the nucleus (blue) of both extracellular and intracellular *T. gondii*. Without permeabilization, the sortilin fluorescence signal was not observed. **B** The fluorescence signal of sortilin (green) overlaps with that of the Golgi apparatus marker GROASP (red) in both intracellular and extracellular parasites. **C** The RH strain of *T. gondii* and 5 μg/ml BFA were co-cultured at 37 °C for 4 h (intracellular and extracellular). The fluorescence signals of sortilin (green) and GROASP (red) in both extracellular and intracellular parasites completely disappeared after BFA treatment

Native *T. gondii* sortilin (TGGT1_290160), with a molecular weight of approximately 120 kDa, was detected by Western blotting using sortilin-specific polyclonal antibodies (Additional file 1: Fig. S1E). Sortilin in *T. gondii* was determined using immunofluorescence. The fluorescence signal of sortilin was observed near the nucleus of *T. gondii* tachyzoites, and the fluorescence signal was observed only after the parasites had been permeabilized, indicating that the protein was localized in intracellular *T. gondii* (Fig. 1A). The localization of sortilin in *T. gondii* was further determined by co-staining with an antibody against the Golgi marker GROASP. The fluorescence signals of sortilin and GROASP exhibited colocalization (Fig. 1B). After treatment of *T. gondii* with brefeldin A (BFA), which specifically disrupts the Golgi apparatus, the fluorescence signal of sortilin disappeared (Fig. 1C). These data suggest that sortilin is localized in the Golgi apparatus of *T. gondii*.

### *Toxoplasma gondii* sortilin interacted with several parasite-derived proteins associated with host cell invasion

**Fig. 2:**
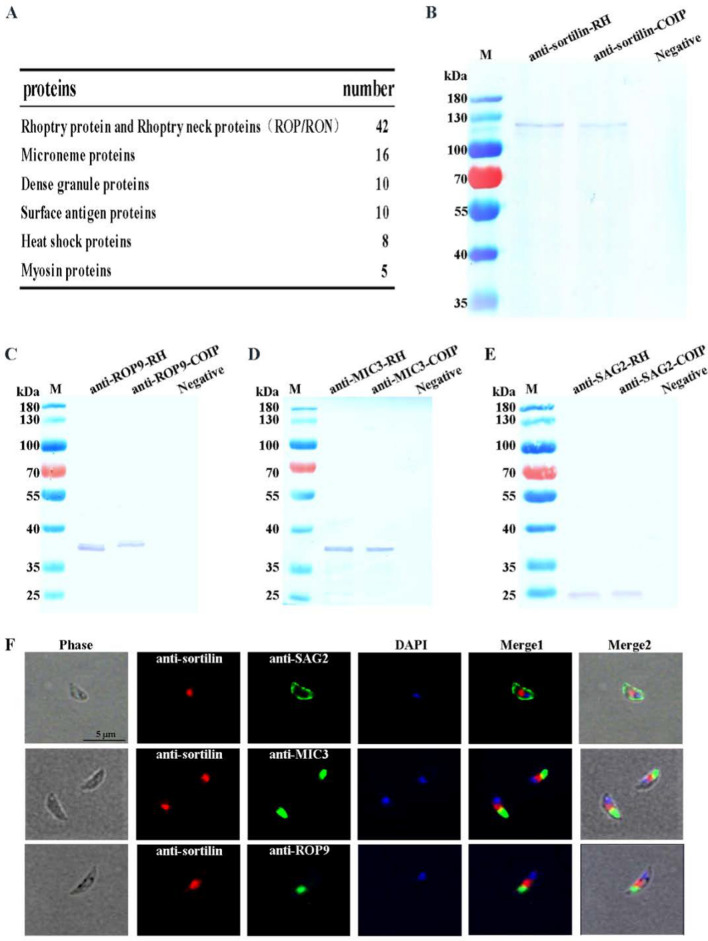
Immunoprecipitation validation of sortilin-interacting proteins. **A** *Toxoplasma gondii* proteins pulled down by the sortilin-specific antibody. **B**–**E** Confirmation of TgROP9, TgMIC3, and TgSAG2 co-precipitation with sortilin by respective protein-specific antibodies in Western blotting. Immunoprecipitates were detected with anti-TgROP9, anti-TgMIC3, and anti-TgSAG2 antibodies. *Toxoplasma gondii* total proteins were used as a positive control. Vero cell total proteins were used as a negative control. **F** Co-localization of TgROP9, TgMIC3, and TgSAG2 (green) with sortilin (red) in *T. gondii*

One hundred *T. gondii* proteins were detected by mass spectrometric analysis of *T. gondii* proteins pulled down using sortilin-specific antibodies (Fig. 2A). Proteins from the SAG, ROP, and MIC families were pulled down using sortilin-specific antibodies (Fig. 2A). The three proteins, TgSAG2, TgROP9, and TgMIC3, in the immunoprecipitates were further confirmed by Western blotting using TgSAG2-, TgROP9-, and TgMIC3-specific antibodies (Fig. 2B–E). Furthermore, sortilin partially co-localized with TgSAG2, TgROP9, and TgMIC3 in the immunofluorescence assays (Fig. 2F).

### Interactions between *T. gondii* sortilin and ROP9, MIC3, and SAG2 were determined by BLI

**Fig. 3:**
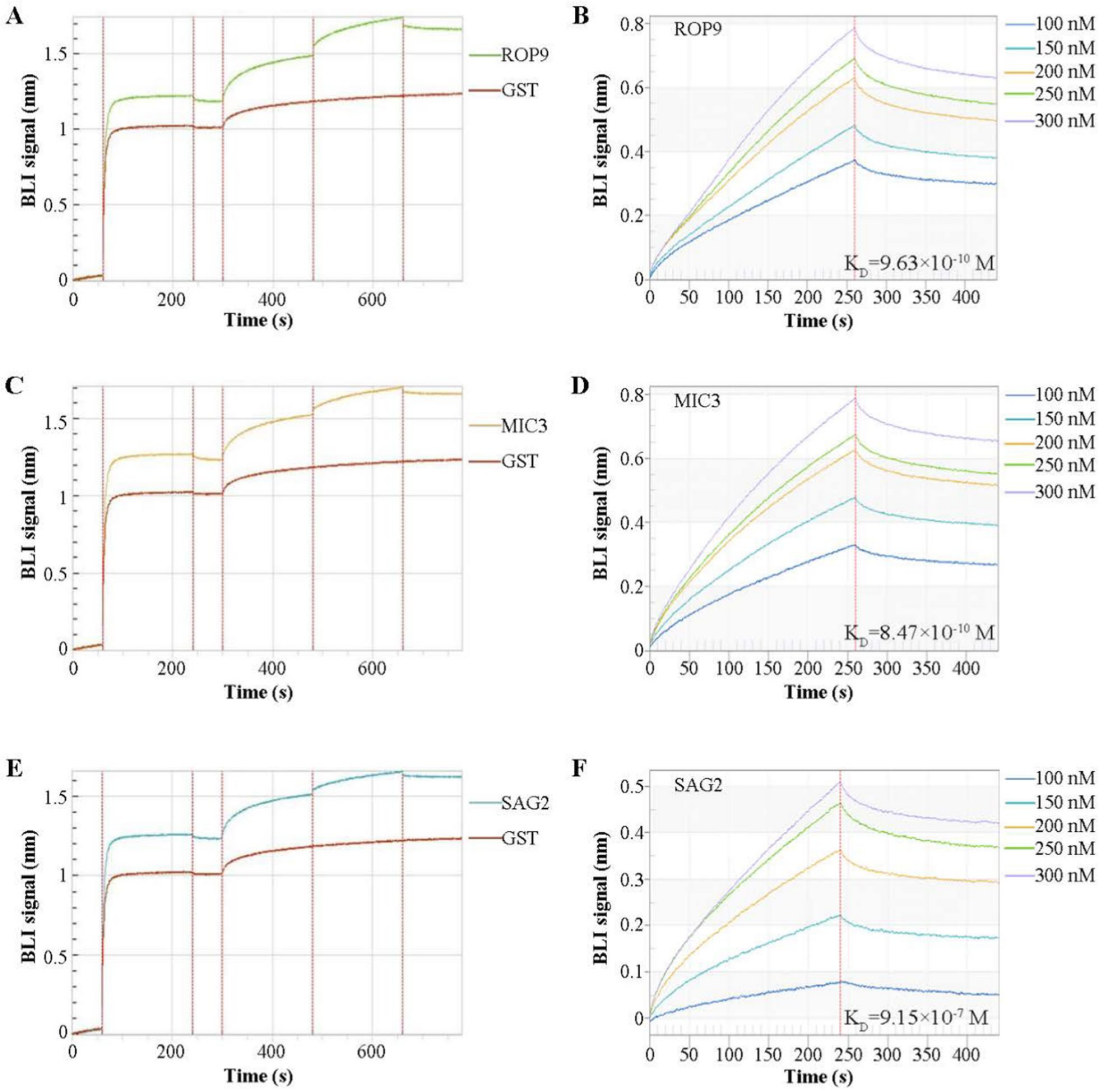
Interactions between sortilin and TgROP9, TgMIC3, and TgSAG2 were analyzed using BLI. **A**–**F** Interaction of recombinant sortilin with TgROP9, TgMIC3, and TgSAG2 was verified by BLI. Sortilin interacted with TgROP9, TgMIC3, and TgSAG2 in a concentration-dependent manner. The GST protein was used as a control

The molecular interactions among sortilin and TgROP9, TgMIC3, and TgSAG2 were investigated using a molecular interaction analyzer. Strong interactions were observed between sortilin and TgROP9, TgMIC3, and TgSAG2. The binding between sortilin and TgROP9, TgMIC3, and TgSAG2 increased as the concentration of sortilin recombinant protein increased (Fig. 3A–F). GST protein was used as a negative control.

### The Vps10, sortilin-C, and sortilin-M subdomains were found to interact with TgROP9, TgMIC3, and TgSAG2

**Fig. 4:**
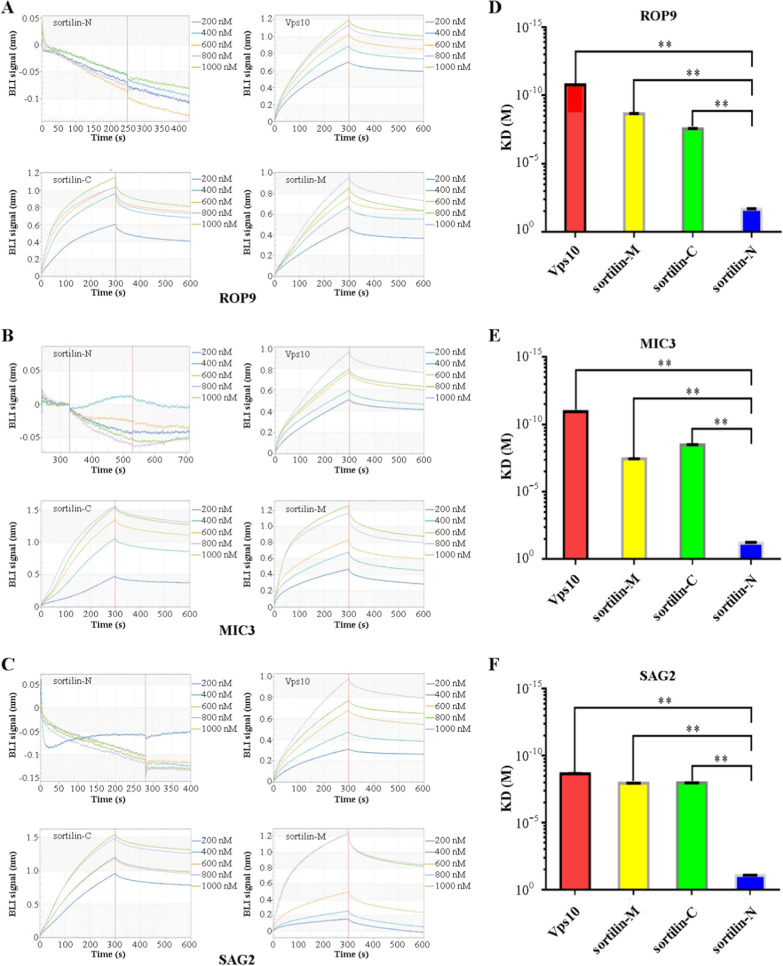
Characterization of the binding of the subdomains (sortilin-N, Vps10, sortilin-C, and sortilin-M) with TgROP9, TgMIC3, and TgSAG2. **A**–**C** The interaction of sortilin subdomains (Vps10, sortilin-C, sortilin-M) of *T. gondii* with TgROP9, TgMIC3, and TgSAG2 was concentration dependent. **D**–**F** Vps10, sortilin-C, and sortilin-M showed significant affinity for TgROP9, TgMIC3, and TgSAG2, but sortilin-N did not. Error bars represent the mean ± SD (*n* = 3). **P < 0.01

Molecular interaction experiments of TgROP9, TgMIC3, and TgSAG2 with GST-tagged recombinant sortilin-N, Vps10, sortilin-C, and sortilin-M showed that Vps10, sortilin-C, and sortilin-M subdomains could bind to TgROP9, TgMIC3, and TgSAG2 with high affinity, whereas GST-sortilin-N did not show any affinity for TgROP9, TgMIC3, or TgSAG2 (Fig. 4A–F). The GST protein was used as a control.

### The sortilin inhibitor AF38469 inhibited the invasion, proliferation, and infectivity of *T. gondii*

**Fig. 5:**
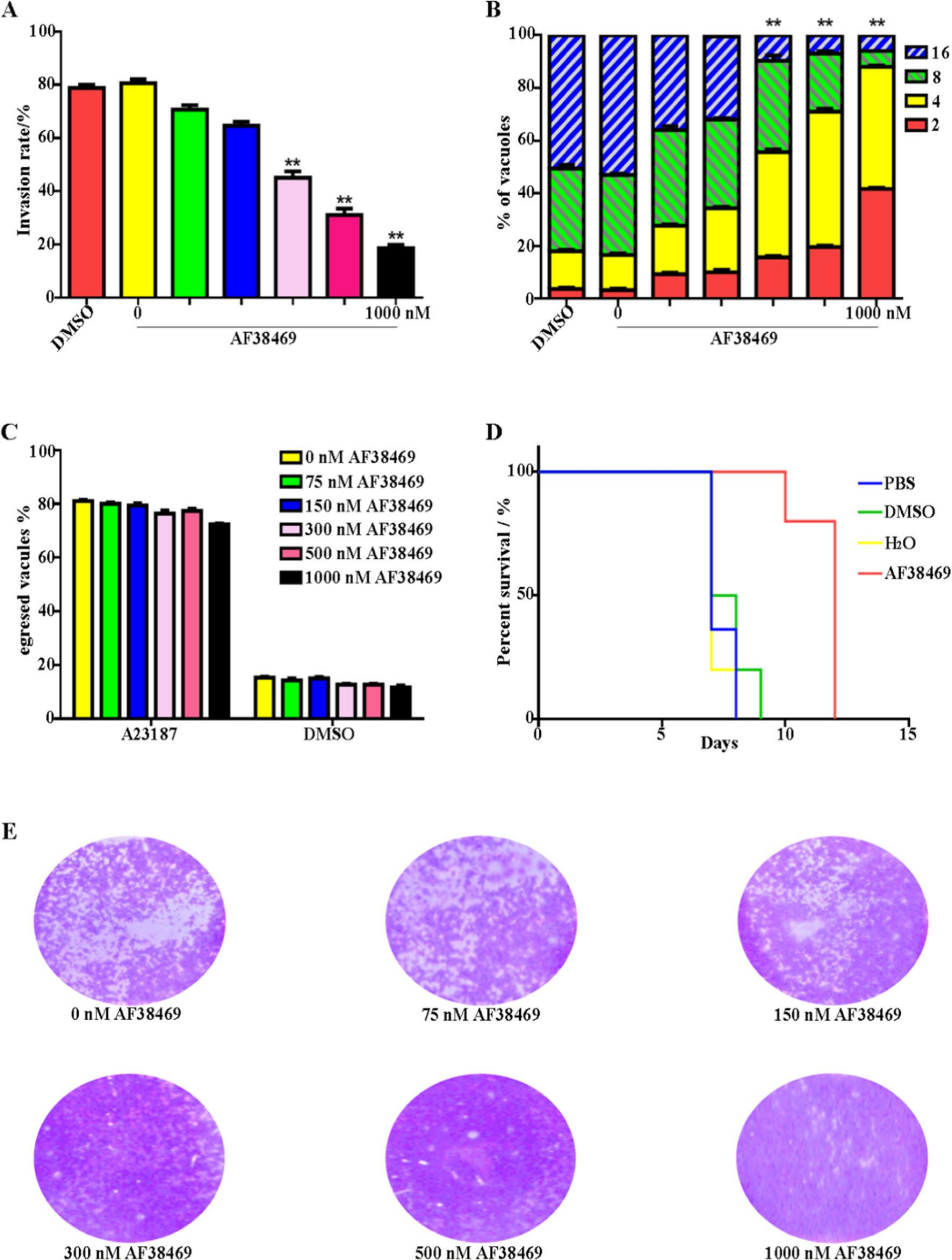
The sortilin inhibitor AF38469 inhibited parasite invasion, proliferation, and infectivity of *T. gondii.* **A**–**C** *Toxoplasma gondii* invasion and replication rates were reduced by AF38469 in vitro, but the egress rate was not affected. Drug concentrations were set to 0, 75, 150, 300, 500, and 1000 nM. DMSO was used as a negative control. Error bars represent the mean ± SD (*n* = 3). **P < 0.01. **D** Mouse survival time was prolonged by oral administration of 500 nM AF38469. Each mouse was infected intraperitoneally with 100 *T. gondii* tachyzoites. DMSO and PBS were used as negative controls. **E** *Toxoplasma gondii* intracellular growth was inhibited by AF38469 in plaque assays. Drug concentrations were set to 0, 75, 150, 300, 500, and 1000 nM

The sortilin inhibitor AF38469 has a molecular formula of C_15_H_11_F_3_N_2_O_3_ and a molecular weight of 324.25 g/mol (Additional file 1: Fig. S4). The effects of gradient concentrations of AF38469 on *T. gondii* invasion, intracellular replication, and egress were verified using red/green assays. The inhibitor concentrations were 0, 75, 150, 300, 500, and 1000 nM. *Toxoplasma gondii* invasion and replication rates were reduced by AF38469 in vitro; however, the egress rate was not affected (Fig. 5A–C).

Each mouse was infected intraperitoneally with 100 *T. gondii* tachyzoites. Prolonged mouse survival was observed after oral administration of 500 nM AF38469 (Fig. 5D). The plaque assay showed that the intracellular growth of *T. gondii* was inhibited by treatment with different concentrations of AF38469 (Fig. 5E).

### The Vps10 subdomain was found to interact with the sortilin inhibitor AF38469

**Fig. 6:**
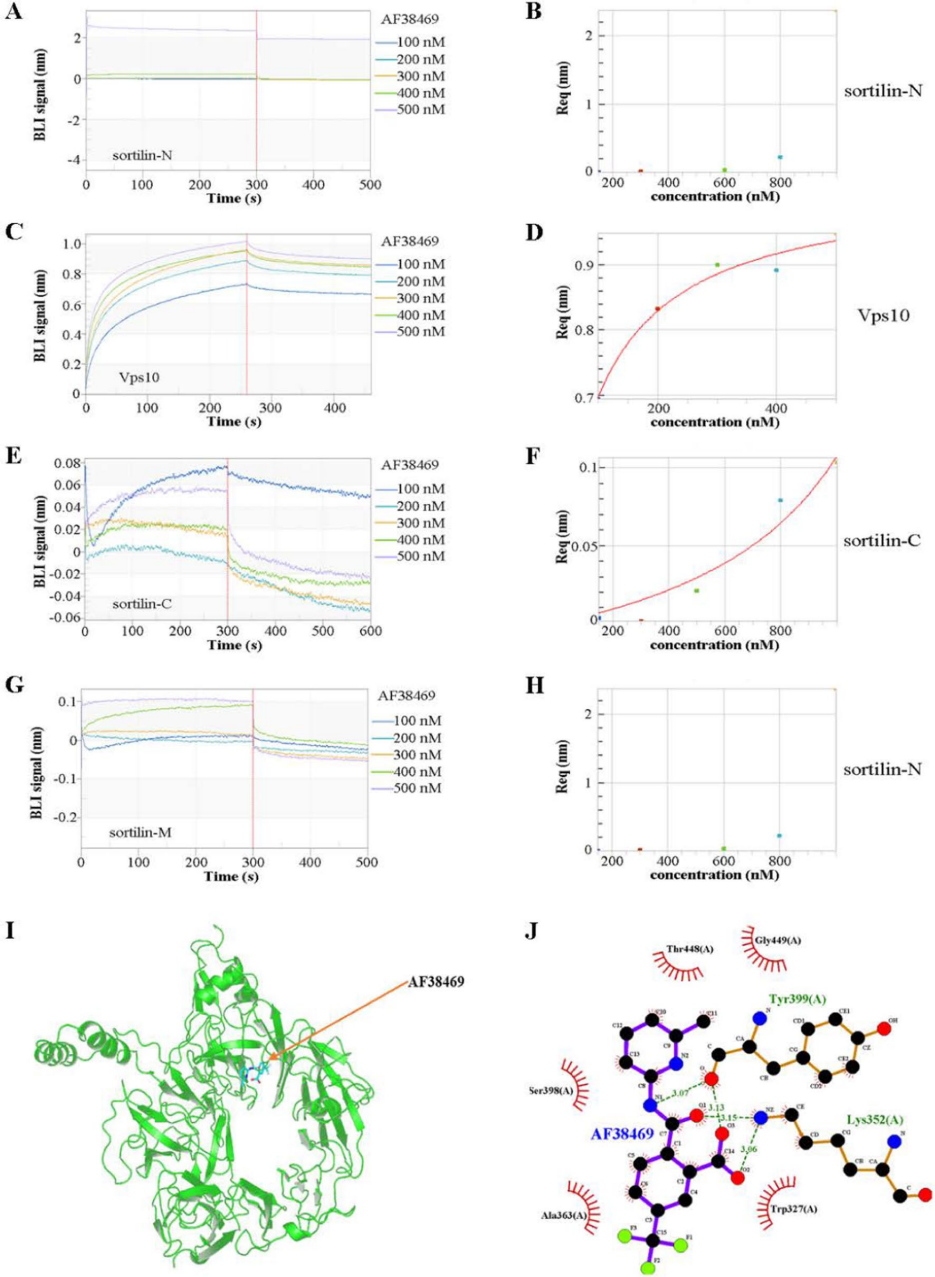
The Vps10 subdomain was found to interact with the sortilin inhibitor AF38469. **A**–**H** The Vps10 subdomain showed a significant affinity for AF38469 in a concentration-dependent manner and formed a better static curve, but sortilin-N, sortilin-C, and sortilin-M did not. DMSO was used as a negative control. **I** Binding position of the inhibitor AF38469 (red arrow) to the Vps10 subdomain (green) in the 3D structure. **J** The simulated binding site of the inhibitor AF38469 to the Vps10 subdomain was located at lysine (Lys352) and tyrosine (Tyr399)

The binding region between the sortilin inhibitor AF38469 and sortilin was investigated using a molecular interaction analyzer. Molecular interaction experiments of AF38469 with GST-tagged recombinant sortilin-N, Vps10, sortilin-C, and sortilin-M showed that only the Vps10 subdomain could bind to AF38469 with high affinity in a concentration-dependent manner and form a better static fit curve, whereas the sortilin-N, sortilin-C, and sortilin-M subdomains did not show any affinity for AF38469 (Fig. 6A–H). Simultaneously, the simulation analysis showed that AF38469 was able to bind well to the Vps10 structural domain in the context of the 3D structure (Fig. 6I), and it was likely that AF38469 bound to the lysine (353 aa) and tyrosine (399 aa) sites in the Vps10 structural domain (Fig. 6J).

## Discussion

Toxoplasmosis is a widespread zoonotic parasitic disease for which treatments such as ethidium, pyrimethamine, and sulfasalazine have been used [2, 3]. However, this treatment regimen is limited by the occurrence of several adverse drug reactions. Therefore, *T. gondii* drug targets have become an important area of research. In this study, the Vps10, sortilin-C, and sortilin-M subdomains of *T. gondii* sortilin were identified as functional regions for the intracellular transportation of various host cell invasion-associated proteins. The binding region for the sortilin inhibitor AF38469 was also identified as the Vps10 subdomain. These results suggest that *T. gondii* sortilin is a potential drug target for disease control.

In previous studies, *T. gondii* sortilin (TgSORT, 962 aa) was reported to transport ROP and MIC families to secretory organelles [30]. In this study, *T. gondii* sortilin was composed of 1033 amino acids, 71 amino acids more than that of the previous study. Sequence comparisons suggested that these 71 amino acids were located at the N-terminal end of sortilin. *Toxoplasma gondii* sortilin contains two functional domains, Vps10 (163–665 aa) and sortilin-C (668–848 aa), with a transmembrane region (859–879 aa) but no signal peptide (Additional file 1: Fig. S1A). A comparison of the 3D structure of *T. gondii* sortilin with that of mouse and human sortilin proteins revealed that they possess similar 3D structures and may share similar protein transport functions (Additional file 1: Fig. S1B–D).

The expression of *T. gondii* sortilin was verified by Western blotting, and its localization in the Golgi apparatus was confirmed by immunofluorescence analysis using a sortilin-specific antibody (Fig. 1). Furthermore, the protein could not be identified in the Golgi apparatus after the cells were treated with BFA, which specifically disrupted the organelles. As a transmembrane domain was identified in *T. gondii* sortilin (Additional file 1: Fig. S1A), it is likely that the protein is anchored on the membrane of the Golgi apparatus with a function in protein trafficking. Immunoprecipitation assays with sortilin-specific antibodies revealed that many *T. gondii* proteins, including ROP, MIC, and SAG family proteins, were pulled down (Fig. 2A). Furthermore, the co-localization of sortilin with TgROP9, TgMIC3, and TgSAG2 was detected by immunofluorescence and confirmed by BLI (Figs. 2F and 3), further supporting the association of sortilin with ROP9, MIC3, and SAG2 proteins.

To determine which of the sortilin subdomains was involved in the protein transport process, the molecular interactions of sortilin subdomains with TgROP9, TgMIC3, and TgSAG2 were further analyzed using BLI techniques. The results showed that the Vps10, sortilin-C, and sortilin-M subdomains could bind to TgROP9, TgMIC3, and TgSAG2 in a concentration-dependent manner; however, the sortilin-N subdomain showed no binding affinity (Fig. 4).

We further confirmed that sortilin is a promising drug target against *T. gondii*. Recent studies have shown that the sortilin inhibitor AF38469 effectively inhibits the invasion of pancreatic and brain cancers [31, 32]. In the present study, AF38469 was found to inhibit *T. gondii* invasion and intracellular replication. Moreover, the survival of *T. gondii*-infected mice treated with AF38469 was prolonged (Fig. 5). BLI and AlphaFold2 machine learning analyses indicated that the binding site of AF38469 in sortilin is located in the Vps10 subdomain, with specific interactions with lysine (Lys352) and tyrosine (Tyr399) residues (Fig. 6).

## Conclusion

In conclusion, the *T. gondii* sortilin, located in the Golgi apparatus of the parasite, can transport a variety of proteins associated with host cell invasion, such as ROP, MIC, and SAG family proteins. The Vps10, sortilin-C, and sortilin-M subdomains of *T. gondii* sortilin were identified as functional regions for intracellular protein transport. The binding region for the sortilin inhibitor AF38469 was also identified as the Vps10 subdomain. This study establishes sortilin as a promising drug target against *T. gondii* and provides a valuable reference for the development of anti-*T. gondii* drug-target studies. The findings of this study could potentially lead to the development of novel therapeutic agents for the treatment of *T. gondii* infections, which remains a significant global health burden.

### Supplementary Information

**Additional file 1: Table S1.** The primers used in this study. **Figure S1.** Structural analysis of sortilin and its expression in *Toxoplasma gondii*. (A) The analysis revealed that sortilin contains two structural domains, namely Vps10 and sortilin-C (blue), and a transmembrane region (green). (B) Simulated 3D structure of *T. gondii* sortilin. (C) Comparison of the 3D structural simulation of *T. gondii* sortilin (red) with that of mouse sortilin (green). (D) Comparison of the 3D structural simulation of *T. gondii* sortilin (red) with that of human sortilin (blue). (E) Western blot analysis of native sortilin expression in both ME49 and RH strains of *T. gondii* detected by a sortilin-specific antibody. Healthy rat serum was used as a negative control. **Figure S2.** Validation of sortilin recombinant proteins by Western blot. (A) His-tagged sortilin recombinant protein was detected by Western blot. (B) GST-tagged sortilin recombinant protein was detected by Western blot. **Figure S3.** The recombinant proteins of TgROP9, TgMIC3, TgSAG2, sortilin-N, Vps10, sortilin-C, and sortilin-M were identified by Western blotting. (A) Sortilin was subdivided into four domains: sortilin-N (white region), Vps10 (red region), sortilin-C (blue region), and sortilin-M (yellow region). (B) Recombinant proteins of GST-sortilin-N, GST-Vps10, GST-sortilin-C, and GST-sortilin-M were detected by Western blotting with a GST-specific antibody. (C) Recombinant proteins of His-TgROP9, His-TgMIC3, and His-TgSAG2 were detected by Western blotting with a HIS-tag specific antibody. **Figure S4.** Molecular diagram of the inhibitor AF38469.

## Data Availability

All data will be available on request after the manuscript is published.

## Abbreviations

BLI: Bio-layer interferometry
BFA: Brefeldin A
ROPs: Rhoptry proteins
RONs: Rhoptry neck proteins
MICs: Microneme proteins
SAGs: Surface antigen proteins
GRAs: Dense granule secreted proteins
ER: Endoplasmic reticulum
PV: Parasitophorous vacuole
MJ: Moving junction structure
SORT1: Sortilin
NTSR-3: Neurotensin receptor-3
AP: Alkaline phosphatase
ELISA: Enzyme-linked immunosorbent assay
PFA: Paraformaldehyde
DMSO: Dimethyl sulfoxide
FBS: Fetal bovine serum
