# Supplementary material for: Toxoplasma sortilin interacts with secretory proteins and it is critical for parasite proliferation
Source: Parasit Vectors. 2024 Mar 4;17:105. doi: 10.1186/s13071-024-06207-7 (PMC10910794; doi:10.1186/s13071-024-06207-7)
Supplement: Supplementary file 1 — Additional file 1: Table S1. The primers used in this study. Figure S1. Structural analysis of sortilin and its expression in Toxoplasma gondii. (A) The analysis revealed that sortilin contains two structural domains, namely Vps10 and sortilin-C (blue), and a transmembrane region (green). (B) Simulated 3D structure of T. gondii sortilin. (C) Comparison of the 3D structural simulation of T. gondii sortilin (red) with that of mouse sortilin (green). (D) Comparison of the 3D structural simulation of T. gondii sortilin (red) with that of human sortilin (blue). (E) Western blot analysis of native sortilin expression in both ME49 and RH strains of T. gondii detected by a sortilin-specific antibody. Healthy rat serum was used as a negative control. Figure S2. Validation of sortilin recombinant proteins by Western blot. (A) His-tagged sortilin recombinant protein was detected by Western blot. (B) GST-tagged sortilin recombinant protein was detected by Western blot. Figure S3. The recombinant proteins of TgROP9, TgMIC3, TgSAG2, sortilin-N, Vps10, sortilin-C, and sortilin-M were identified by Western blotting. (A) Sortilin was subdivided into four domains: sortilin-N (white region), Vps10 (red region), sortilin-C (blue region), and sortilin-M (yellow region). (B) Recombinant proteins of GST-sortilin-N, GST-Vps10, GST-sortilin-C, and GST-sortilin-M were detected by Western blotting with a GST-specific antibody. (C) Recombinant proteins of His-TgROP9, His-TgMIC3, and His-TgSAG2 were detected by Western blotting with a HIS-tag specific antibody. Figure S4. Molecular diagram of the inhibitor AF38469. [file 13071_2024_6207_MOESM1_ESM.docx]

**Additional file 1**

**Table. S1.** The primers used in this study.

| **Primers** | **sequence (5’-3’)** |
| --- | --- |
| sortilin-F | GGATCCGTTCTGGTTGCTAACG |
| sortilin-R  sortilin-N-F  sortilin-N-R | CTCGAGTTAAGACACGGGAG  ATGTACACCTCTCGTACCCCG  TCAACGGGTTTTCAGCAGGAT |
| Vps10-F | CGTGGCCGTCTGTACC |
| Vps10-R | GATAACGATGTTGTCAACGAACAGC |
| sortilin-C-F | GAACCGAACGCTAGCAGC |
| sortilin-C-R | CGGACATGGCACCGC |
| sortilin-M-F  sortilin-M-R  ROP9-F  ROP9-R  MIC3-F  MIC3-R  SAG2-F  SAG2-R | TTGAAAAAATTCTTCCGTAACGCG  TTACAGCAGTTCAACGTTATCTTCG  ATGAGTTCTTCCAATTTTAGGG  TCACTGCATGATCAACGA  ATGCGAGGCGGGACGT  TCACTGCTTAATTTTCTCACACG  ATGAGTTTCTCAAAGACCACGAGC  TTACACAAACGTGATCAACAAACCT |

**
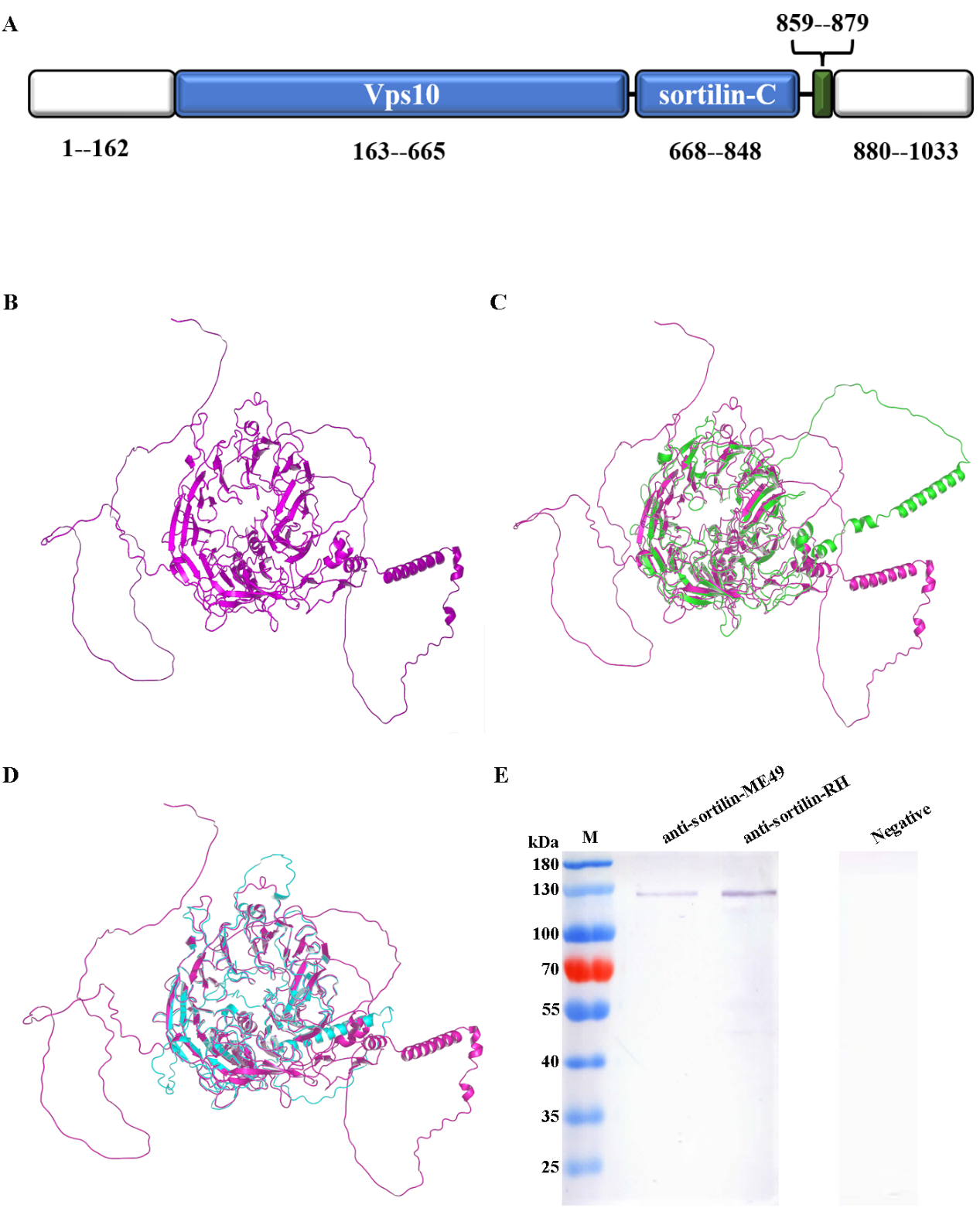
**

**Figure S1.** **Structural analysis of sortilin and its expression in *T. gondii*.** (A) The analysis revealed that sortilin contains two structural domains, namely Vps10 and sortilin-C (blue), and a transmembrane region (green). (B) Simulated 3D structure of *T. gondii* sortilin. (C) Comparison of the 3D structural simulation of *T. gondii* sortilin (red) with that of mouse sortilin (green). (D) Comparison of the 3D structural simulation of *T. gondii* sortilin (red) with that of human sortilin (blue). (E) Western blot analysis of native sortilin expression in both ME49 and RH strains of *T. gondii* detected by a sortilin-specific antibody. Healthy rat serum was used as a negative control.


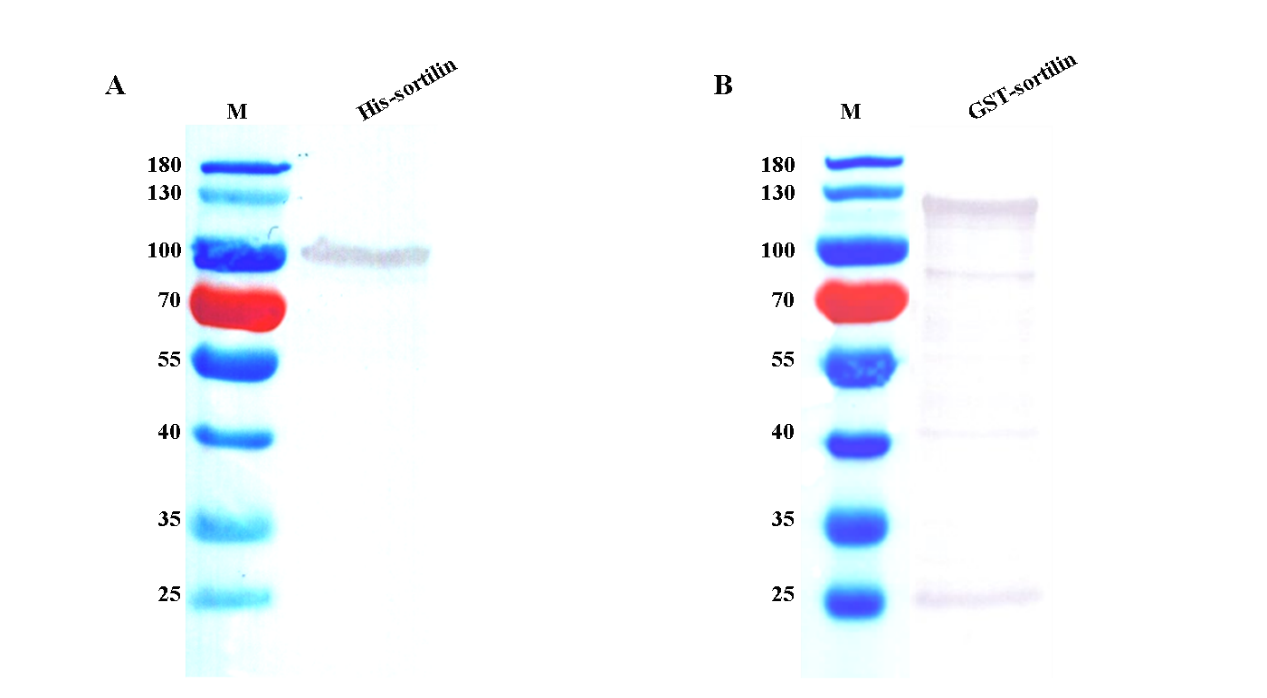


**Figure S2. Validation of sortilin recombinant proteins by Western blot.** (A) His-tagged sortilin recombinant protein was detected by Western blot. (B) GST-tagged sortilin recombinant protein was detected by Western blot.

**Generation of recombinant TgROP9, TgMIC3 and TgSAG2**

The coding gene fragments of TgROP9 (gene ID: TGGT1_243730), TgMIC3 (gene ID: TGGT1_319560), and TgSAG2 (gene ID: TGGT1_208850) were cloned into the pET-28a and pGEX-4T-1 expression vectors after PCR amplification using gene-specific primers (Table S1). The two recombinant plasmids were expressed in E. Coli BL21 (DE3) cells (TransGen Biotech, Beijing, China). His- and GST-tagged recombinant proteins were obtained by affinity purification, as described previously [33].

**Generation of specific antibodies to TgROP9, TgMIC3 and TgSAG2**

Specific polyclonal antibodies were obtained by immunising mice and rats with His-tagged recombinant proteins of TgROP9, TgMIC3 and TgSAG2. Eight female BALB/c mice and three female Sprague-Dawley rats were subcutaneously immunized four times with Freund’s adjuvant. Each mouse was immunized subcutaneously with 50 μg of the HIS-tagged recombinant protein each time, and each rat was immunized subcutaneously with 100 μg of the recombinant protein each time. Blood was collected from the hearts of the rats and eyes of the mice. Blood samples were incubated at 37°C for 1 h and then centrifuged at 3500 rpm for 10 minutes to separate the sera. All sera were inactivated at 56°C for 30 min. Rat and mouse anti-TgROP9, TgMIC3 and TgSAG2 IgGs were purified from serum using Protein G Sepharose 4 Fast Flow (GE Healthcare, Chicago, IL, USA), as described previously [33].


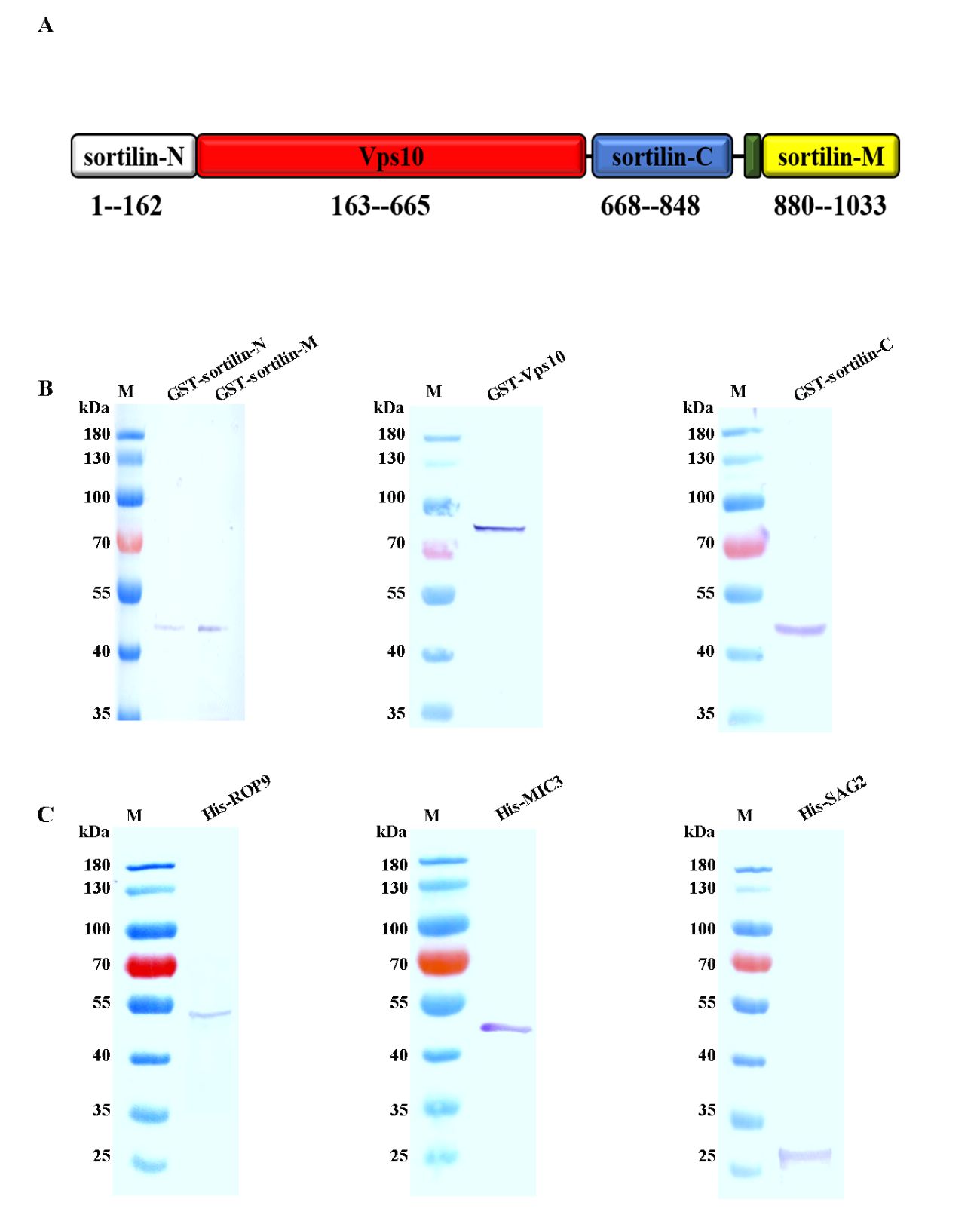


**Figure S3. The recombinant proteins of TgROP9, TgMIC3, TgSAG2, sortilin-N, Vps10, sortilin-C, and sortilin-M were identified by western blotting.** (A) Sortilin was subdivided into four domains: sortilin-N (white region), Vps10 (red region), sortilin-C (blue region), and sortilin-M (yellow region). (B) Recombinant proteins of GST-sortilin-N, GST-Vps10, GST-sortilin-C, and GST-sortilin-M were detected by western blotting with a GST-specific antibody. (C) Recombinant proteins of His-TgROP9, His-TgMIC3, and His-TgSAG2 were detected by western blotting with a HIS-tag specific antibody.


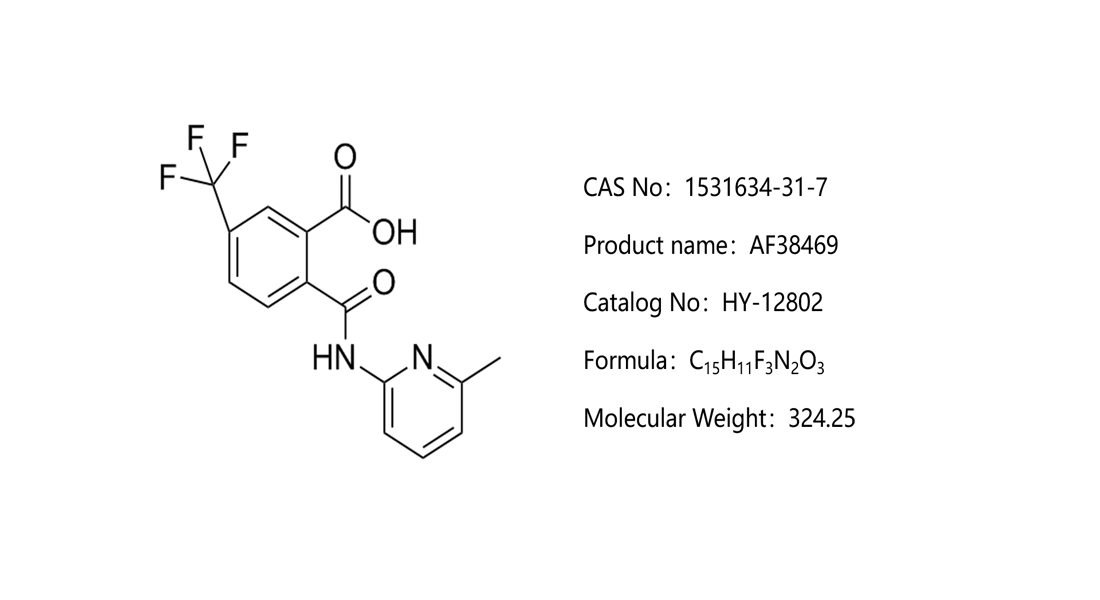


**Figure S4. Molecular diagram of the inhibitor AF38469.**
